# Beyond immunomodulation: mechanisms and synergistic strategies of mesenchymal stem cells in promoting alveolar epithelial and endothelial repair in ARDS

**DOI:** 10.3389/fimmu.2026.1769714

**Published:** 2026-06-12

**Authors:** Zhuowei Yu, Ting Zhang, Zhangxue Hu, Mi Yan, Jia Tang

**Affiliations:** Department of Pediatrics, Daping Hospital, Army Medical University, Chongqing, China

**Keywords:** alveolar epithelial repair, ARDS, immunomodulation, MSCs, pulmonary endothelial repair, synergistic therapeutic strategies

## Abstract

ARDS is a life-threatening lung disease marked by severe AECS and ECs damage, causing rapid lung function loss and high mortality. MSCs are promising therapies due to their immunoregulatory and regenerative properties. While early studies highlighted MSCs’ anti-inflammatory effects in ARDS, recent findings emphasize their essential role in repairing the AECs and ECs. In this review, we detail the molecular mechanisms and synergistic effects of MSCs in epithelial and endothelial repair. We also examine how MSC-mediated immune regulation intersects with tissue repair, explore new combination approaches using drugs, gene editing, and bioengineering, and outline clinical limitations and ongoing challenges. Patient heterogeneity, disease stage, treatment timing, and safety issues limit the conduct of clinical trials and their translation. We summarize the roles, limitations, and challenges of MSCs in ARDS to guide future research and inform the development of new treatments.

## 1 Introduction

**Figure 1:**
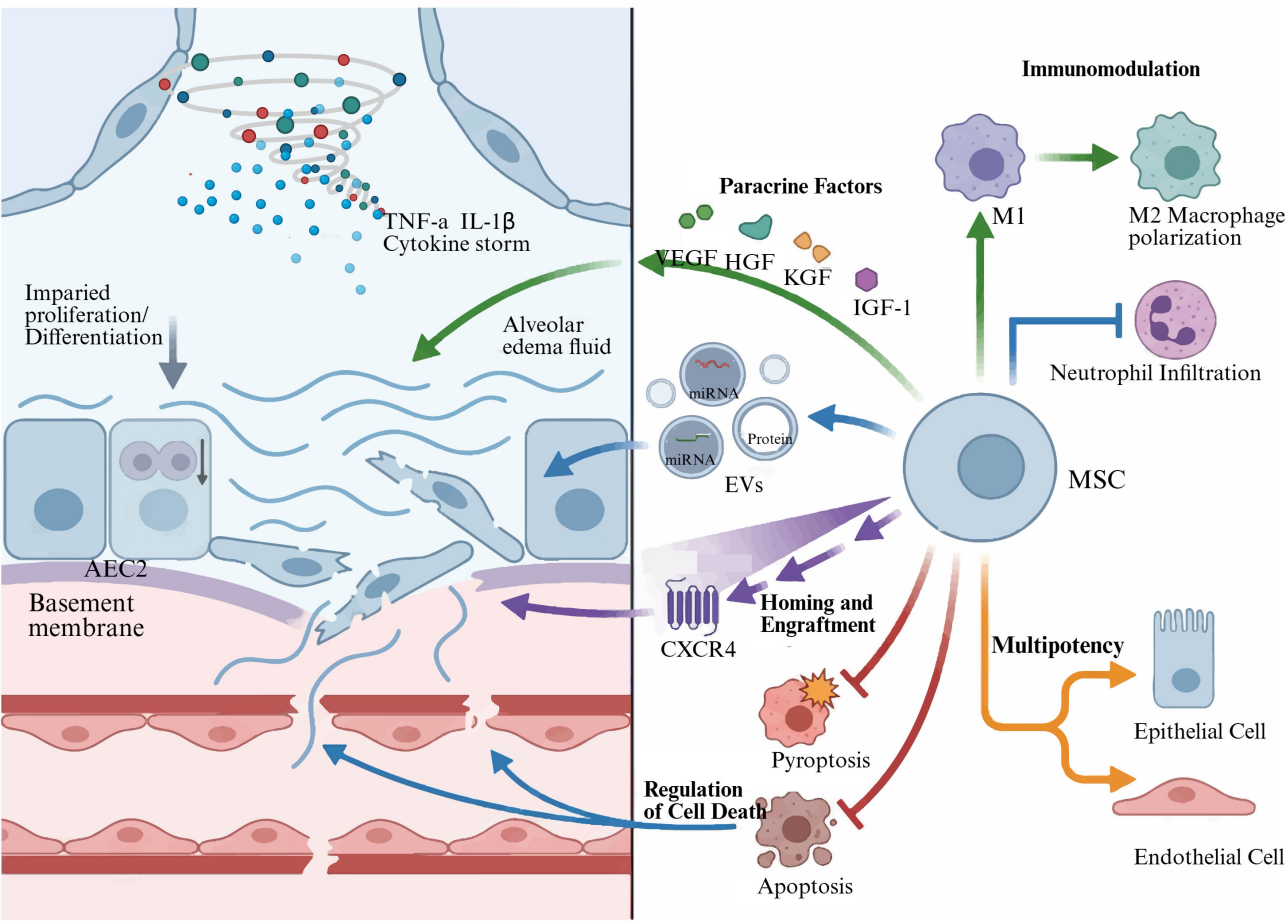
MSCs target multiple pathophysiological hallmarks of ARDS. The injured alveolar-capillary unit in ARDS is characterized by diffuse damage to AECs and pulmonary ECs, resulting in disruption of tight junctions, alveolar edema, and a cytokine storm. Impaired differentiation of AECs 2 progenitors into functional AEC1 cells further hinders barrier regeneration. MSCs exert therapeutic effects through a coordinated array of mechanisms: 1) Paracrine secretion of growth factors (VEGF, HGF, KGF, IGF-1); 2) Immunomodulation by polarizing macrophages to an anti-inflammatory M2 phenotype and restraining neutrophil infiltration; 3)EV-mediated communication carrying proteins, lipids, and regulatory miRNAs; 4)Homing and colonization to injury sites guided by CXCR4; 5)Multidirectional differentiation potential under specific microenvironmental cues; and 6)Regulation of programmed cell death in AECs. Figure created with BioRender.com and obtained relevant publishing licenses.

ARDS is a severe clinical syndrome with acute respiratory failure and diffuse lung inflammation, and high mortality despite better supportive care (1). Its key pathological features are widespread injury to AECs and ECs, which damage the alveolar-capillary barrier, increase fluid leakage, and lead to pulmonary edema (2, 3). Barrier breakdown leads to severe edema, impaired gas exchange, and persistent hypoxemia (4, 5). Pathogenesis involves uncontrolled inflammation, oxidative stress, cell death, and failed repair (6–8). Cytokines and chemokines shape ARDS, and immune cell interactions with AECs and ECs are crucial to disease progression and resolution (**Figure 1**) (9–11).

Current ARDS treatments depend on supportive ventilation and fluid management, with no effective drugs that directly promote lung repair or restore the barrier (12–14). The lung’s regenerative capacity is under active study, and AECs 2’ role as progenitors in alveolar repair is notable (15, 16). In severe ARDS, incomplete maturation of AECs 1 may result in persistent barrier dysfunction.

MSCs offer promise for ARDS therapy through their multipotency, differentiation potential, immunomodulatory effects, and broad paracrine effects. They secrete reparative factors (17–19), modulate immunity, reduce inflammation, stimulate epithelial and endothelial repair, and improve alveolar fluid clearance. These actions address multiple mechanisms of ARDS (20–24). MSCs’ paracrine effects, mediated by EVs containing proteins, nucleic acids, and mitochondria, drive immunomodulation and regeneration (25). EVs can restore mitochondrial function in damaged AECs and ECs, improving barrier function. MSCs regulate immunity and also enhance direct repair of AECs and ECs. They upregulate tight junction proteins, strengthening the alveolar-capillary barrier.

Although MSCs have shown promise in ARDS treatment, challenges remain in clinical application. Factors such as patient variability, disease staging, timing, and safety considerations limit trial design and translation. This review summarizes the complex roles, limitations, and challenges of MSCs in ARDS to guide research and inspire new treatment strategies.

## 2 Methods

This review was conducted using a systematic search across PubMed, Embase, Scopus, the core collection of Web of Science, and CNKI, covering the period from [January 2015] to [December 2025]. Additionally, to obtain the latest research progress, the preprint platforms medRxiv and bioRxiv were consulted. The English search term combinations used include: “metabolic stem/strategic cells”; (“acute respiratory distress syndrome” or “ARDS”); and (“epithelial repair” or “essential repair” or “immune modulation”). Inclusion criteria: preclinical and clinical studies (including randomized controlled trials, observational studies, and meta-analyses) focusing on the role of MSCs in the repair mechanisms of the alveolar epithelium or pulmonary ECs in ARDS, as well as important reviews. Literature screening was independently completed by two authors, and differences were resolved through discussion.

## 3 The mechanisms underlying MSCs’ role in repairing lung injury caused by ARDS

### 3.1 Common mechanisms in ARDS lung injury repair

MSCs can differentiate into various cell types, regulate immune responses, and aid in repairing lung tissue after ARDS. They interact with the extracellular matrix, sensing and responding to mechanical signals, adjusting their metabolism, releasing helpful factors, changing the environment around stem cells, and influencing how cells develop and function (26).

#### 3.1.1 Paracrine effect: molecular basis for repair signals

MSCs release a mixture of beneficial molecules that signal to nearby cells. This process helps control inflammation, supports new blood vessel growth, encourages cell survival and division, and prevents cell death. Important factors such as VEGF and HGF help form new blood vessels, bringing the oxygen and nutrients needed to damaged lung tissue in ARDS. When VEGF and HGF act in concert, they enhance this effect. IGF-1 and FGF also help various lung cell types survive, grow, and move by triggering specific signals within cells (27) (**Figure 1**).

#### 3.1.2 Immune regulatory function: cultivating a repair-conducive immune microenvironment in ARDS

A primary mechanism by which MSCs repair is by regulating the immune response at the injury site (21). They shift the body’s response from inflammation to healing. MSCs do this by direct contact with immune cells and by releasing soluble factors. They weaken overactive immune responses, limit harmful M1-type macrophages, and reduce excess neutrophil activity (28). MSCs also influence dendritic cells by reducing their ability to activate other immune cells. The main molecules involved in this immune control include IDO, PGE2, TGF-β, and IL-10, all of which promote an environment that supports tissue repair rather than inflammation.

#### 3.1.3 EVs-mediated intercellular communication

EVs are nanoscale membrane vesicles secreted by cells. They mediate intercellular communication by transporting substances. MSC-derived EVs deliver their cargo to target cells, influencing the structure and function of those cells (28–30). The extent to which EVs can move and interact with other cells depends on cell density. In both living organisms and laboratory conditions, most EVs are quickly taken up by neighboring cells, making them primarily short-range communication messengers. Their effects are typically restricted to the tissues or organs where they were produced (31). This localized action highlights the crucial role of EVs in regulating their immediate environment, particularly during repair processes associated with ARDS. EVs derived from MSCs are not only carriers of intercellular communication, but also the core hub connecting immune regulation and tissue repair. The multiple bioactive molecules carried by EVs, such as miRNAs, achieve synergistic regulation of immune and ECs functions. At the level of immune regulation, miRNAs such as miR-21-5p and miR-146a, which are abundant in MSC-derived EVs, are taken up by alveolar macrophages and promote macrophage polarization from the pro-inflammatory M1 phenotype to the anti-inflammatory M2 phenotype by targeting signaling pathways, thereby creating a favorable immune microenvironment for tissue repair. At the level of endothelial repair, miR-126 carried by MSC-derived EVs promotes ECs proliferation and migration by activating the MAPK/ERK signaling pathway, while miR-148a-3p enhances the expression of tight junction protein ZO-1 by regulating the ROCK1 pathway, directly improving alveolar capillary barrier function. More importantly, there is positive feedback between EV-mediated immune regulation and endothelial repair: factors secreted by M2 macrophages can further enhance the endothelial repair capacity of MSC-derived EVs and the repaired endothelial barrier can limit excessive infiltration of inflammatory cells, forming a virtuous cycle. This networked regulatory mechanism suggests that MSC-derived EVs are not isolated parallel interactions, but rather achieve integrated regulation of the immune microenvironment and vascular barrier function through miRNAs, providing a theoretical basis for EV-based engineering modifications (**Figure 1****).**

#### 3.1.4 Homing and colonization: precise navigation to injured sites

The homing of MSCs to injured sites is a meticulously regulated, multi-step process, primarily driven by the interaction between chemokine receptors expressed on the surface of MSCs and chemokine signals released by the injured microenvironment. In addition to chemokine receptors, adhesion molecules play a crucial role in the rolling, adhesion, and trans-endothelial migration of MSCs along the vascular wall. Upon successful homing to the injured site, the survival and functional maintenance of MSCs in the harsh local microenvironment are pivotal for their therapeutic efficacy. Nevertheless, the long-term colonization rate of MSCs at the injured site remains low. They are more likely to function as “transient” cells, initiating endogenous repair mechanisms in the host through potent paracrine actions, EV release, and other mechanisms in the short term, before undergoing apoptosis or being cleared (32).

#### 3.1.5 Multidirectional differentiation potential: cellular basis for structural reconstruction

Although MSCs possess multilineage differentiation potential, their primary mechanism in repairing ARDS-induced lung injury is not direct differentiation to replace damaged cells, but rather paracrine signaling and intercellular communication that support the repair and regeneration of endogenous cells (32, 33). *In vivo* tracing studies have demonstrated that intravenously administered MSCs are predominantly retained in the pulmonary capillary bed and rapidly cleared, with long-term colonization rates at injury sites of <1%-5%, indicating that direct differentiation contributes minimally to structural reconstruction. The more established mechanism involves MSCs secreting KGF and Wnt signaling molecules to stimulate the proliferation of endogenous AEC2 and their differentiation into AEC1 (34–36).

While MSCs can differentiate into functional cells under specific microenvironmental cues, and their differentiation trajectory may serve as a “local fine-tuning mechanism” providing auxiliary support under certain conditions (34), this is not the central therapeutic pathway in ARDS. Epigenetic modifications, including histone acetylation, regulate MSC differentiation fate decisions, but current evidence primarily derives from *in vitro* chondrogenic and osteogenic studies (33, 34); their specific regulatory roles within the ARDS lung microenvironment require further investigation (**Figure 1**). Thus, the reparative function of MSCs should be understood as an integrated model predominantly governed by paracrine effects, with differentiation playing a supplementary role—paracrine actions not only directly promote tissue repair but also establish a favorable microenvironment for endogenous stem cell activation and integration.

#### 3.1.6 MSCs regulate apoptosis and pyroptosis in ARDS

MSCs regulate the programmed cell death of AECs through multiple pathways, thereby reducing the extent of lung injury in ARDS. MSCs can restore energy metabolism in damaged cells through mitochondrial transfer, and inhibit the activation of apoptotic signals by secreting anti-inflammatory cytokines (21). EVs derived from MSCs have been shown to inhibit NLRP3 inflammasome activation, reduce Gasdermin D-mediated pyroptosis, and protect alveolar epithelial integrity (25). *In vitro* studies also suggest that MSCs may regulate the expression of apoptosis-related genes in target cells by delivering specific miRNAs, as well as exert protective effects by inhibiting endoplasmic reticulum stress pathways. However, direct evidence for these mechanisms in the context of ARDS is not yet sufficient, and further *in vivo* studies are needed to verify them (**Figure 1**).

The role of MSCs in ARDS treatment exhibits clear stage-dependent characteristics, which is of great significance for optimizing clinical treatment strategies. During the acute inflammatory phase (24–72 hours after injury), the pathological features primarily include a cytokine storm, extensive neutrophil infiltration, and acute destruction of the alveolar capillary barrier. At this stage, the dominant repair mechanisms of MSCs are immune regulation and anti-apoptosis: by secreting factors such as PGE2, TSG-6, and IL-10, MSCs induce macrophage polarization towards the M2 phenotype and inhibit excessive inflammatory responses (27). At the same time, through mitochondrial transfer and paracrine HGF pathways, the apoptosis rates of AECs and ECs are significantly reduced, providing a basis for subsequent repair and cell preservation. As the disease progresses to the proliferative repair phase, the pathological focus shifts towards cell proliferation and structural reconstruction, and the therapeutic effect of MSCs correspondingly shifts to promoting endogenous regeneration and vascular stability. At the level of epithelial repair, KGF secreted by MSCs activates the Gab1/ERK/NF - κB signaling axis to promote AEC2 proliferation, while driving AEC2 differentiation towards AEC1 through the Wnt/β - catenin pathway (35, 36). At the level of endothelial repair, activation of the Ang-1/Tie2 signaling pathway enhances vascular barrier integrity. The evolution of the dependency mechanism at this stage suggests that early intervention can maximize the benefits of immune regulation, whereas later treatment requires strengthening the capacity to promote regeneration. For patients with different subtypes and stages of inflammation, MSCs from different tissue sources or optimized through pre-treatment should be selected to achieve precise treatment.

### 3.2 MSCs and alveolar epithelial stem cell interactions

**Figure 2:**
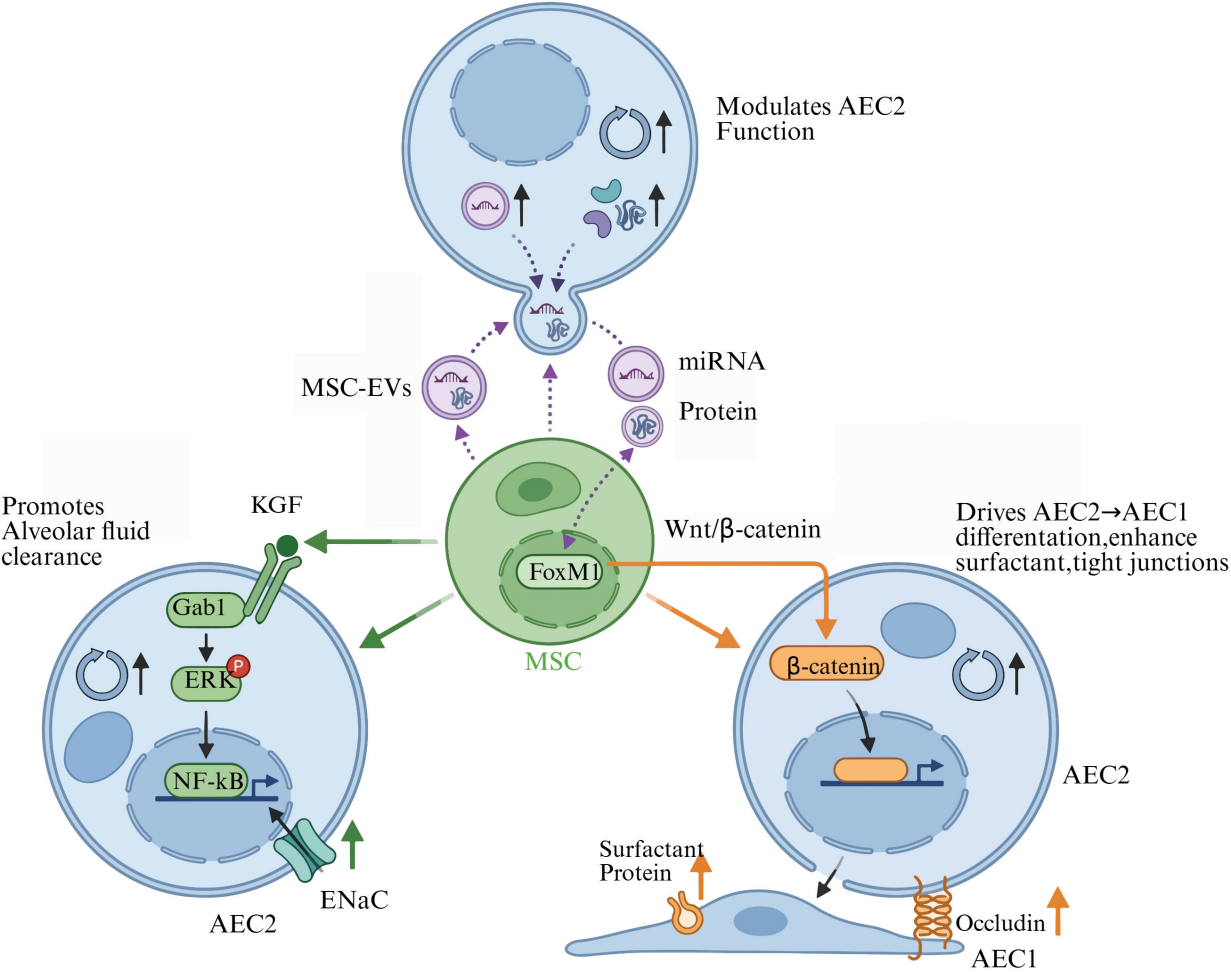
Mechanisms of MSC-mediated alveolar epithelial repair. This figure details the specific molecular mechanisms by which MSCs promote the repair and regeneration of AECs: 1) Secretion of KGF activates the Gab1/ERK/NF-κB signaling axis in AEC2, upregulating ENaC expression to enhance alveolar fluid clearance. 2) Release of MSC-EVs delivers a cargo of miRNAs and proteins that modulate AEC2 gene expression and function. 3) Activation of the Wnt/β-catenin signaling pathway drives the differentiation of AEC2 into AEC1, as evidenced by increased surfactant protein expression and β-catenin nuclear translocation. Figure created with BioRender.com and obtained relevant publishing licenses.

MSCs have been recognized as key regulators of AEC repair, primarily by supporting the activation and differentiation of endogenous alveolar epithelial progenitors rather than by direct differentiation into epithelial cells. KGF secreted by MSCs has been shown to promote AEC2 proliferation and repair by activating the Gab1/ERK/NF-κB signaling axis, thereby upregulating ENaC expression and enhancing alveolar fluid clearance and epithelial barrier function in ALI models (35) (**Figure 2**). MSCs can regulate the differentiation direction of AEC2 at the molecular level through Wnt/β-catenin signaling. Overexpression of the transcription factor FoxM1 in MSCs promotes AEC2-to-AEC1 differentiation through activation of Wnt/β-catenin signaling, as demonstrated by upregulation of surfactant proteins and nuclear translocation of β-catenin (36) (**Figure 2**).

This mechanism does not entail the direct transformation of MSCs into epithelial cells; rather, it drives the targeted differentiation of endogenous alveolar epithelial progenitor cells through paracrine signaling, thereby achieving alveolar epithelial regeneration. This mechanism involves MSCs driving endogenous alveolar epithelial progenitor differentiation via paracrine signals, rather than MSCs themselves differentiating into AECs (**Figure 2**).

### 3.3 Mechanisms of MSCs promoting pulmonary endothelial repair

#### 3.3.1 Regulation of endothelial barrier function and permeability

**Figure 3:**
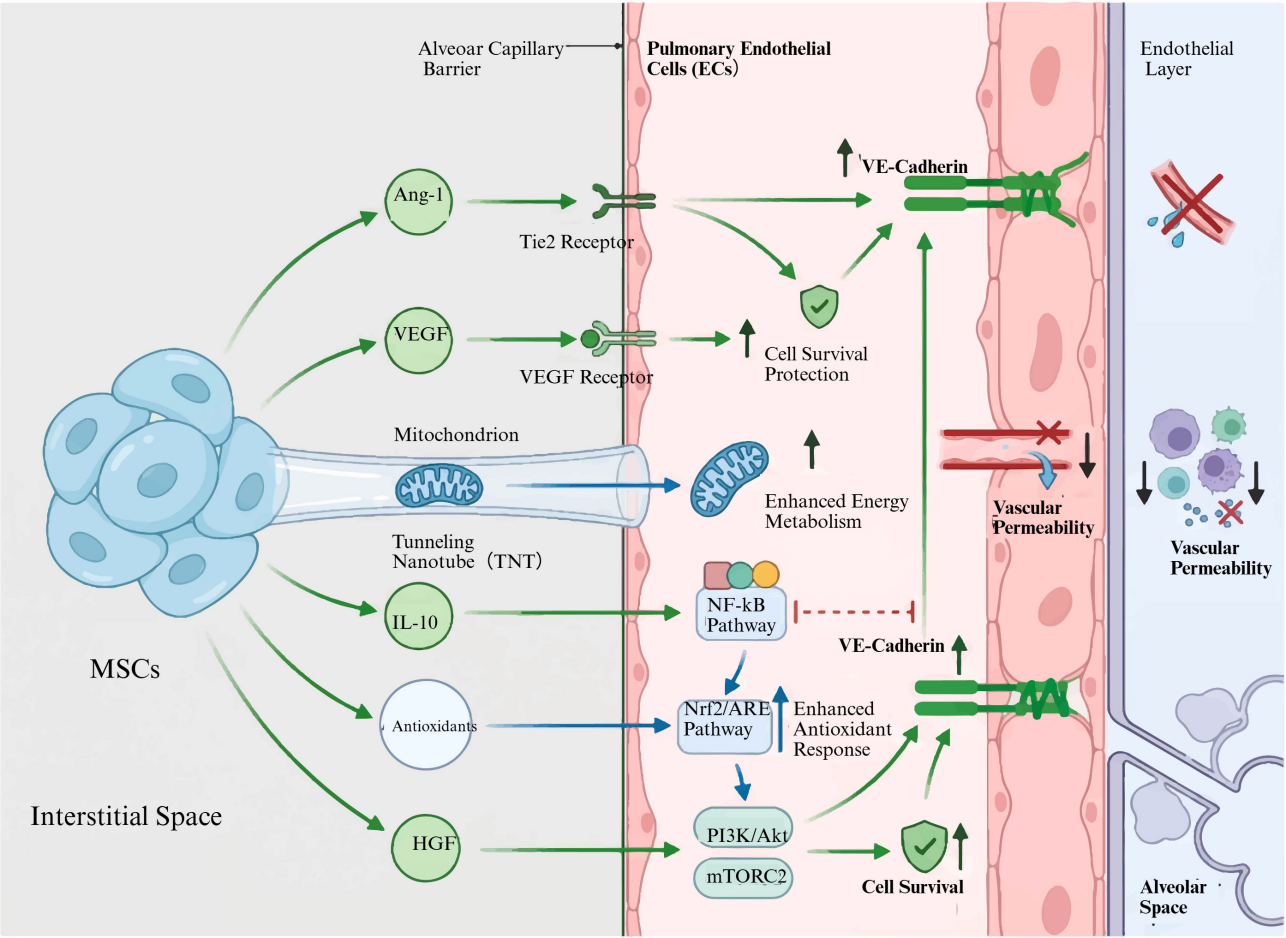
Mechanisms of MSC-mediated pulmonary endothelial stabilization and repair. MSCs engage in multifaceted crosstalk with ECs: 1) Enhancing endothelial junctions. 2)Exerting anti-inflammatory and antioxidant effects. 3)Activating pro-survival and junctional reorganization pathways. Figure created with BioRender.com and obtained relevant publishing licenses.

VEGF and Ang-1 secreted by MSCs can strengthen endothelial cell-cell junctions and facilitate the recovery of pulmonary capillary barrier function. Specifically, Ang-1 binds to the Tie2 receptor on ECs, suppressing inflammatory responses and reducing permeability, thereby protecting adherens junctions from degradation and preserving vascular integrity. Additionally, MSCs can transfer mitochondria to damaged pulmonary microvascular ECs through tunneling nanotubes, enhancing their energy metabolism and sustaining barrier function. MSCs can also promote ECs survival, proliferation, and junctional remodeling by activating the mTORC2 and PI3K/Akt signaling pathways. Factors released by mechanically preconditioned MSCs can inhibit ECs apoptosis and restore intercellular junctions. HGF secreted by MSCs similarly enhances endothelial barrier repair through mTORC2 activation. Direct cell-cell communication via gap junctions—formed by connexin proteins such as Conn43—allows metabolic exchange and promotes anti-inflammatory activity, maintaining endothelial barrier integrity (37). Integrin-mediated adhesion further stabilizes the vascular structure (38). It is crucial to emphasize that the crux of these mechanisms lies in MSCs enhancing the intrinsic function and repair capacity of ECs through paracrine signaling and intercellular communication, rather than directly differentiating into ECs to form vascular structures (**Figure 3**).

#### 3.3.2 Modulation of endothelial inflammation and oxidative stress

MSCs exert potent anti-inflammatory effects on ECs primarily by secreting anti-inflammatory cytokines. These secreted factors modulate endothelial inflammatory signaling pathways, including the NF-κB pathway, attenuating the expression of pro-inflammatory cytokines and adhesion molecules that contribute to endothelial activation and dysfunction (35). In addition to anti-inflammatory actions, MSCs mitigate oxidative stress in ECs by regulating the Nrf2/ARE signaling pathway. This antioxidative mechanism is crucial for preserving endothelial function, as oxidative stress is a major contributor to endothelial barrier disruption and vascular inflammation in ARDS and other inflammatory diseases.

MSCs can be engineered to synergize with nanomaterials or to be genetically modified to enhance their antioxidative and anti-inflammatory capacities. Overexpression of transcription factors, including FoxM1 in MSCs, has also been reported to enhance endothelial proliferation, migration, and barrier integrity by modulating signaling pathways, indirectly reducing oxidative stress and inflammation (36). The paracrine factors secreted by MSCs promote EC proliferation and migration, thereby supporting capillary network formation (**Figure 3**).

#### 3.3.3 Promotion of endothelial proliferation, migration, and angiogenesis

MSCs have been extensively investigated for their ability to enhance EC proliferation and migration, which are prominent steps in vascular repair and regeneration. As detailed in Section 2.1.3, MSC-derived EVs carry a variety of miRNAs that regulate endothelial functions. HUCB-MSCs and their secreted exosomes promoted the proliferation and migration of VE tip cells by downregulating TGF-β1 via exosomal miR-21-5p, reducing apoptosis, and increasing cell viability (38). MSCs might similarly activate the PI3K/AKT and MAPK/ERK signaling cascades to promote ECs’ survival and orchestrate angiogenesis. Wnt5a-overexpressing MSCs exert protective effects on LPS-induced injury in ECs through the PI3K/AKT pathway, promoting migration, proliferation, and angiogenic capacity of ECs while suppressing apoptosis (39). miR-126 delivered by MSCs participates in vascular repair by activating the MAPK/ERK signaling pathway, enhancing endothelial differentiation and limiting neointimal hyperplasia. The proangiogenic activity of MSC-EVs is also mediated by miRNA cargo, including miR-21-5p and let-7c-5p, which promote endothelial regeneration by stimulating proliferation and migration while suppressing apoptosis (40, 41).

Beyond soluble factors, direct crosstalk between MSCs and ECs plays an essential role in vascular repair. MSCs preferentially adhere to injured endothelial monolayers via membrane molecules, facilitating direct cell-cell interactions and downregulating markers of endothelial injury and permeability, thereby increasing wound healing and angiogenic capacity (42, 43). MSC-expressed lncRNAs, such as SCDAL, regulate angiogenesis by activating noncanonical VEGF receptor 2 signaling in ECs through the induction of GDF6 (44, 45). The PTEN/AKT/VEGF axis, targeted by MSC-secreted factors and exosomes, enhances EC survival and angiogenesis (46).

This crosstalk is bidirectional: ECs secrete factors, including VEGFA and PDGF-BB, that maintain MSC stemness, promote differentiation toward pericyte-like phenotypes, and stimulate MSC migration to injury sites, thereby establishing a supportive vascular niche (47). Hypoxic preconditioning of MSCs enhances their angiogenic potential by upregulating pro-angiogenic miRNAs and proteins, which are delivered to ECs via EVs, increasing endothelial proliferation and tube formation (48). Physical interactions via gap junctions and integrin-mediated adhesion reinforce MSC-EC coupling, facilitating metabolic exchange and stabilizing nascent vessels (49). However, the roles of specific miRNAs in endothelial repair have been primarily demonstrated in models of myocardial ischemia, hindlimb ischemia, or *in vitro* HUVEC studies (39, 40, 50). Their specific regulatory functions in pulmonary microvascular ECs within the ARDS microenvironment require further validation.

### 3.4 Comprehensive mechanism of MSCs promoting synergistic repair of AECs and ECs

#### 3.4.1 Immune Regulation alleviates inflammatory environment

As detailed in Section 2.1.2, MSCs modulate immune responses by promoting an anti-inflammatory microenvironment. This immunomodulatory action not only protects AECs and EVs but also creates favorable conditions for their subsequent repair (**Figure 1**). Overall, the immunoregulatory functions of MSCs offer a multifaceted approach to alleviating the inflammatory environment in ARDS. By suppressing excessive inflammation and fostering a regenerative milieu, MSCs not only protect AECs and ECs but also enhance their repair processes, ultimately improving outcomes in patients with ARDS and related inflammatory conditions.

#### 3.4.2 Cell communication and signaling networks

MSCs play a pivotal role in regulating intercellular communication, particularly between epithelial and ECs, by secreting various factors. The paracrine signaling mechanisms employed by MSCs facilitate the transfer of bioactive molecules, which are crucial for maintaining homeostasis and promoting tissue repair in the alveolar environment (51). Furthermore, the interaction between MSCs and target cells is bidirectional and mediated by a complex signaling network that ensures a coordinated response to injury. Key molecules such as FoxM1 and LPA are instrumental in mediating the intricate signaling networks that govern the recovery of cellular function during the reparative processes initiated by MSCs. LPA, a bioactive lipid, has been shown to activate multiple signaling pathways that promote cell survival and migration, facilitating the repair of the alveolar-capillary barrier.

#### 3.4.3 Promoting the recovery of alveolar barrier structure and function

**Figure 4:**
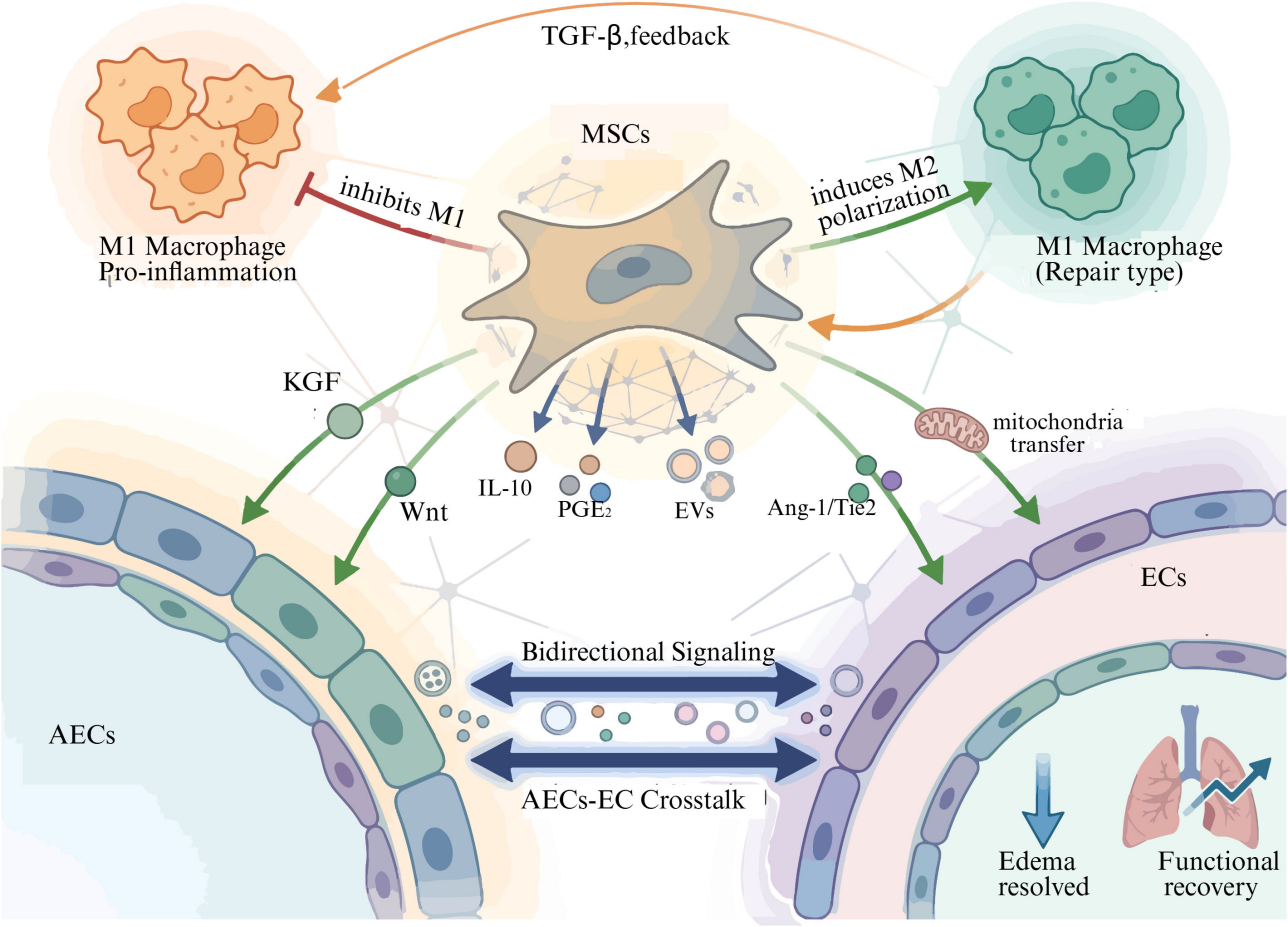
Synergistic crosstalk: MSCs orchestrate alveolar-capillary barrier repair through integrated networks. MSCs shift the immune microenvironment from pro-inflammatory to pro-repair by polarizing M1 macrophages to an M2 phenotype. These M2 macrophages, in turn, secrete factors that provide positive feedback to MSCs and create a regenerative niche. The MSC secretome simultaneously acts on both parenchymal cell types, promoting alveolar epithelial repair and endothelial stabilization. Bidirectional crosstalk between the repaired epithelium and endothelium is also facilitated. Figure created with BioRender.com and obtained relevant publishing licenses.

The synergistic effects of MSCs on epithelial regeneration and endothelial repair are crucial in mitigating ALI/ARDS. As discussed in Sections 2.2 and 2.3, MSC-derived factors simultaneously promote AEC proliferation (via KGF/Wnt) and EC stabilization (via Ang-1/Tie2), leading to coordinated restoration of the alveolar-capillary barrier (21, 52, 53). This dual action, together with the immunomodulatory effects described in Section 2.1.2, underscores the integrated therapeutic potential of MSCs (**Figure 4**).

#### 3.4.4 Promoting the remodeling of the pulmonary microenvironment

MSCs are critical for remodeling the pulmonary microenvironment, particularly in ARDS. This is particularly crucial in ARDS, where the existing vasculature may be compromised due to inflammation and injury. The interplay between MSCs and these cells can lead to a more robust and sustained repair process, facilitating the restoration of normal lung function over time. MSCs can enhance the proliferation and differentiation of AECs, which are crucial for re-establishing the alveolar-capillary barrier, often disrupted in ARDS (54). Moreover, the immunomodulatory properties of MSCs can help mitigate the inflammatory response characteristic of ARDS, fostering a more favorable microenvironment for tissue repair. This dual action—both structural and immunological—underscores the potential of MSCs not only to repair but also to optimize the lung microenvironment, ultimately improving clinical outcomes in patients with ARDS. MSCs play a pivotal role in remodeling the pulmonary microenvironment by regulating ECM composition and enhancing angiogenesis.

### 3.5 Bidirectional feedback between immunomodulation and tissue repair

The therapeutic effect of MSCs in ARDS is greatly enhanced by bidirectional crosstalk between immunoregulatory and tissue-repair processes. MSCs’ potential to reprogram the immune microenvironment supports reparative activities. MSCs control the immune response by inducing a shift in macrophage polarization from pro-inflammatory M1 to anti-inflammatory M2, which, in turn, decreases pro-inflammatory cytokines, and increases production of anti-inflammatory mediators. This immunomodulatory property creates a more conducive environment for tissue repair by mitigating the cytokine storm and suppressing the migration of immune cells into the lung parenchyma, (55, 56). During the reparative phase, MSCs contribute to the regeneration of AECs and ECs by secreting growth factors and EVs that promote cell proliferation, migration, and differentiation. And, those regenerative activities are bidirectional: the cytokines and growth factors released to repair tissues continue to shape the immune response. Paramount among these factors is HGF, secreted by MSCs, which not only supports epithelial repair but also exerts immunomodulatory effects by modulating pro-inflammatory responses and promoting vascularity (57, 58).

This bidirectional interaction represents a positive feedback loop, in which the immunomodulatory state induced by MSCs shapes their potential to participate in tissue repair, and repair-related cytokines and cellular crosstalk tailor immune responses. Such mutual feedback enhances the therapeutic effect of MSCs in ARDS. The paracrine secretome of MSCs is important in this crosstalk; MSC-derived EVs can transport bioactive molecules that modulate immune cell phenotypes (59) and facilitate repair of the alveolar-capillary barrier (60). This interconnected relationship also extends to other stem/progenitor cells, including EPCs, whose survival and function are promoted by MSC-derived factors and which contribute to vascular repair and immune homeostasis (**Figure 4**).

Understanding and harnessing this bidirectional feedback loop can inform the development of optimized MSC-based therapies, including strategies such as cytokine priming, genetic modification, and biomaterial scaffolds to enhance MSC retention and function in injured lung tissue. The dual regulatory relationship between immune modulation and tissue repair, orchestrated by MSCs, represents a pivotal axis for the effective treatment of ARDS and offers promising avenues for translational research and clinical application. (**Figure 4**).

## 4 MSCs combined therapy strategies and their prospects in ARDS

### 4.1 MSCs and combined drug therapy

Combining MSCs with pharmacological agents represents a promising strategy to enhance therapeutic outcomes in ARDS. MSCs possess intrinsic immunomodulatory and regenerative properties that can be potentiated when combined with drugs targeting inflammation and oxidative stress. Glucocorticoids, a class of potent anti-inflammatory agents, have been widely used to suppress excessive inflammatory responses in ARDS. When combined with MSCs, glucocorticoids not only mitigate inflammation but also synergistically promote tissue repair and regeneration within the injured lung microenvironment. This dual action is crucial because suppression of inflammation alone may not be sufficient to restore alveolar epithelial and endothelial integrity; MSC regenerative capacity complements this by facilitating repair processes (61, 62). Importantly, combined therapy enhances the overall efficacy of ARDS treatment by not only dampening harmful immune responses but also by actively promoting alveolar epithelial and endothelial regeneration. The clinical translation of MSCs combined with drugs requires careful consideration of dosing, timing, and delivery methods to maximize synergy while minimizing adverse effects.

### 4.2 MSCs and gene editing technologies combined

The CRISPR/Cas9 approach has recently become a potent and accurate means of genetically editing MSCs to increase their release of reparative molecules or to enhance their protective effects on AECs and ECs that suffer severe damage in ARDS (63, 64). This immune escape function is essential in ARDS, as allogeneic MSCs have been used, and immune rejection would limit therapeutic persistence. Manipulating ncRNAs in MSCs using the CRISPR/Cas9 system can modulate their secretome and enhance their antifibrotic effect. This strategy could be used to platform MSC paracrine actions for lung repair in ARDS. The application of CRISPR/Cas9 also extends to enhancing MSC differentiation and regenerative capacity. Crucially, gene editing could enhance MSCs’ ability to home to and migrate into lung tissue following injury. Improving MSC homing to the lung via gene editing may enhance their therapeutic effects in ARDS by increasing local cell density in alveolar lesions.

**Figure 5:**
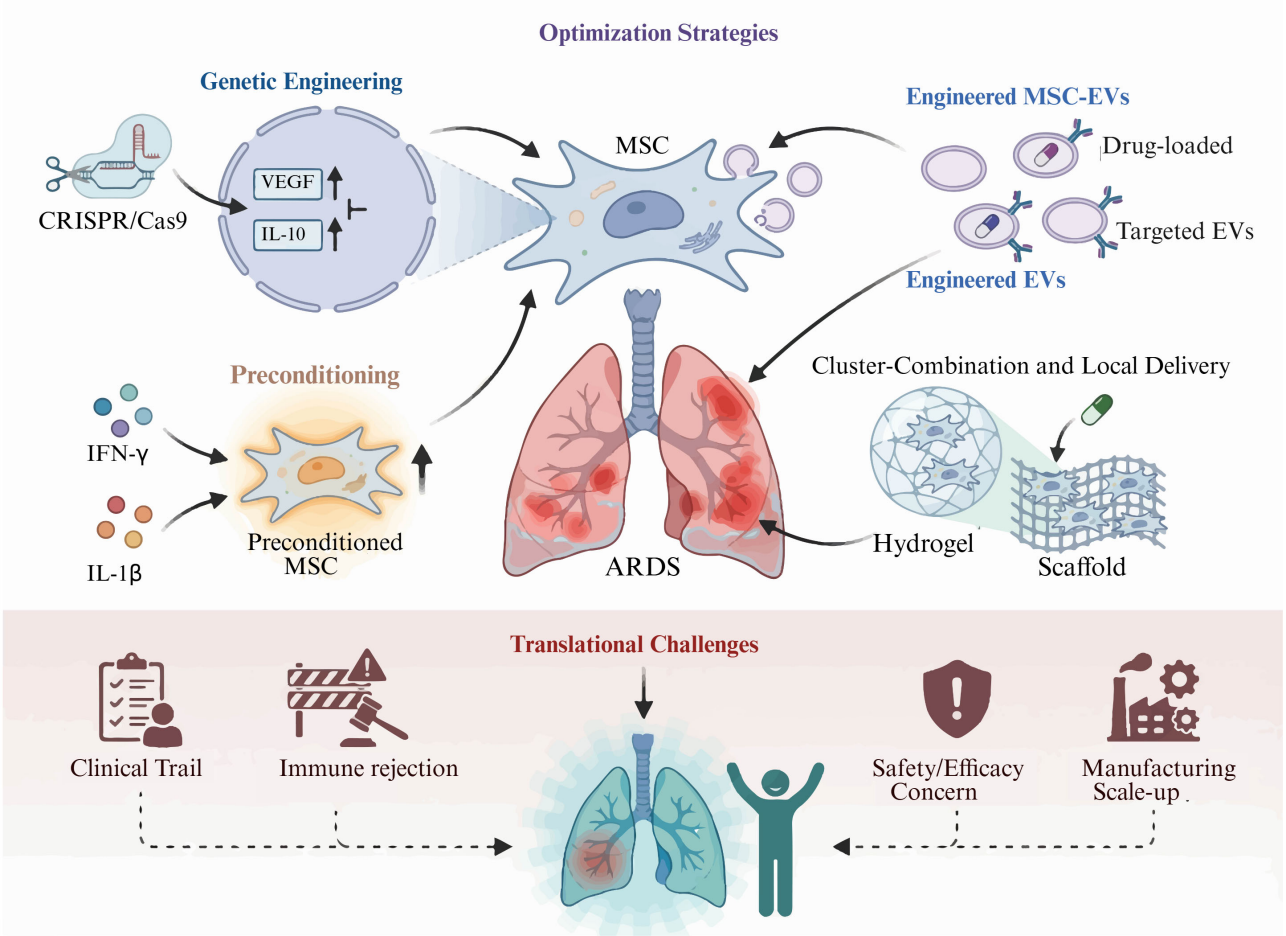
Engineering the future: optimization strategies and translational challenges of MSC therapy for ARDS. Native MSCs can be engineered or combined to augment their potency: 1) Genetic Editing. 2) Engineered EVs. 3) Preconditioning. 4) Combination Strategies. Translational Hurdles: Critical barriers include: 1) Cellular Heterogeneity. 2) Poor *In Vivo* Persistence: Low rates of homing, engraftment, and long-term survival at injury sites. 3) Standardization Hurdles. 4) Patient & Disease Heterogeneity. 5) Long-term Safety Concerns. Figure created with BioRender.com and obtained relevant publishing licenses.

The integration of gene editing with an advanced delivery system and a preconditioning strategy is also shaping the therapeutic efficacy of MSC-based therapies. Nanoparticle magnetization facilitated MSCs targeting and retention in fibrotic lung tissue, enhancing therapeutic effects (65, 66). Combining these targeting strategies with gene editing to augment the secretion of reparative factors could provide synergy in treating ARDS (67, 68). However, it is important to note that current evidence is derived exclusively from *in vitro* and animal model studies; clinical data on safety and efficacy are lacking. Critical issues requiring resolution before clinical translation include off-target effects, long-term genotoxicity, and manufacturing scalability (69, 70). These challenges underscore the need for rigorous preclinical safety assessment and the development of standardized protocols. (**Figure 5**).

### 4.3 MSCs and EVs therapy

MSC-EVs play an essential role in influencing alveolar epithelial stem cell function. These EVs carry cargo that can be delivered to AECs, modulating their functions. MSCs and AECs crosstalk via EVs, which play an essential role in alveolar repair. MSC-EVs therapy as a treatment option for ARDS is versatile and efficacious, promoting lung tissue regeneration, attenuating the inflammatory response, and reconstituting alveolar-capillary barrier function, with an encouraging clinical future. This direct flow of information is reinforced by the release of EVs and soluble factors by MSCs, which share bioactive molecules, such as characteristic miRNAs, proteins, and growth factors, with ECs, influencing their activities. Hypoxic preconditioned MSCs-EVs enhance EC proliferation, migration, and tube formation by targeting the expression of angiogenesis-associated miRNAs and by carrying ECM-receptor interaction-related proteins and Focal Adhesion Proteins in ischemic models, leading to improved microvascular remodeling and neurological outcomes (71). The glycosylation of MSC-EVs, especially N-glycans, is important for their uptake by ECs and is enhanced upon functional activation, underscoring the role of post-translational modification in MSCs-ECs communication (72).

Outstanding questions include identifying the ideal EV source, considering long-term biodistribution, and characterizing mechanisms within a complex ARDS microenvironment. Factors of the lung microenvironment, such as ECs-derived EVs, can influence MSC therapeutic potential through metabolic pathways, underscoring the need to consider microenvironmental crosstalk in clinical translation (73). Innovative approaches like engineering EV cargo to augment reparative miRNAs or proteins, preconditioning MSCs with disease-derived EVs to amplify regenerative potential, and delivering EVs using biomaterial scaffolds are being explored to achieve optimal therapeutic effects (74–80).

### 4.4 Personalized therapy and optimization of combined strategies

Personalized MSC treatment tailored to the patient’s pathological status represents a fundamental breakthrough, improving therapeutic efficacy for ARDS and related lung pathologies (72, 81–83). Variability in MSC efficacy and engraftment has been observed in clinical trials, including REMODEL and REMEDY, which emphasizes the importance of precision medicine strategies that select the cell source, dose, and timing based on patient pathology. Patients with predominant inflammatory phenotypes may be best served by treatment with MSCs that carry highly immunomodulatory secretomes, whereas those with profound epithelial barrier damage may require a more engineered approach in which MSCs are used to promote epithelial repair (84, 85).

Additionally, the new biomaterial carriers are promising for the MSC delivery and relocation to damaged lung tissue (86). Loading bioactive molecules, like miRNAs, into scaffolds can augment MSC anti-inflammatory and tissue-remodeling activities by directly modulating signaling pathways that initiate inflammation and tissue repair.

Beyond new biomaterials, AI and machine learning platforms used in MSCs therapy also have the potential to improve treatment personalization by predicting optimal cell characteristics and treatment conditions based on patient bioinformatic data. AI-powered algorithms will integrate multi-omic profiles and clinical features to profile MSC populations that are more therapeutically advanced and to help design “combinational therapies”. The precision-based methodology will be essential for addressing key issues, such as variability in MSC potency and inconsistent clinical outcomes. Patient-specific pathological characterization, genetic and pharmacological modifications, and innovative biomaterial delivery systems constitute a comprehensive approach to enhancing MSC-based therapies for ARDS (64).

## 5 Challenges and future research directions of MSCs therapy for ARDS

### 5.1 Safety and efficacy issues of MSCs therapy

Clinical trials investigating MSC therapy for ARDS have progressed from the initial exploratory phase to a larger-scale validation phase, with numerous studies assessing its safety and preliminary efficacy. A meta-analysis of randomized controlled trials included 13 studies and 655 patients. The findings revealed that, compared with the control group, MSC treatment did not increase the risk of adverse events and may even reduce mortality among patients with ARDS (87) (**Table 1**). However, the efficacy and predictive value of alveolar biomarkers still require further large-scale, standardized, randomized controlled trials for confirmation (88). Besides, not all studies have yielded positive outcomes. A prospective, double-blind, multicenter, randomized, placebo-controlled phase IIb clinical trial enrolled 120 patients with ARDS requiring mechanical ventilation, of whom the majority (84%) had COVID-19-related ARDS. The study demonstrated that a single infusion of bone marrow-derived MSCs (10 x 10^6^ cells/kg) did not exhibit significant physiological or clinical benefits compared to placebo, including improvements in oxygenation index or reductions in mortality at 14, 28, 60, or 180 days (99). This suggests that MSC efficacy may be influenced by patients’ baseline biological characteristics, disease phenotypes, or treatment regimens, and that future trials should incorporate biomarkers to identify patient subgroups more likely to benefit (100).

**Table 1:** Summary of clinical evidence for MSC therapy in ARDS.

Study type/trial name	Patient population (n) & etiology	MSC source & intervention	Key findings (efficacy & safety)	Interpretation/note	Reference (in manuscript)
Meta-analysis of RCTs	ARDS patients (N = 655 across 13 studies); mixed etiology.	Various sources (BM, UC, AD); doses varied (typically 1–10×10^6^ cells/kg).	**Safety:** No increased risk of adverse events compared to control. **Efficacy:** Potential reduction in mortality.	Provides strong evidence for the overall safety of MSC therapy and suggests a potential survival benefit.	(87, 88)
Phase IIb RCT (STAT/REMEDY Trial)	Moderate-to-severe ARDS (N = 120, 84% COVID-19-associated).	Bone marrow-derived MSCs; single IV infusion (10×10^6^ cells/kg predicted body weight).	**Efficacy:** No significant benefit in oxygenation or mortality at 14–180 days vs. placebo. **Safety:** Well-tolerated.	The negative outcome highlights the impact of patient heterogeneity, emphasizing the need for future trials to stratify patients or use biomarkers to identify responders.	Described in Section 4.1
Systematic Review/Meta-analysis	ARDS & COVID-19 patients across multiple trials.	Heterogeneous sources (BM-, UC-, AT-MSCs).	**Safety:** Generally well-tolerated. **Efficacy:** Benefits are modest and inconsistent.	Variability in efficacy is largely due to heterogeneity in cell sources, preparation, dosing, and patient populations, with standardization being a key current challenge.	(89–91)
Clinical Trials Overview (Phase I/II)	ARDS patients (primarily early-phase trials).	Diverse sources and regimens.	**Safety:** Good short-term safety profile. **Efficacy & Gaps:** Possible short-term mortality benefit; long-term Efficacy And large-scale evidence still lacking.	Preliminary evidence supports safety, but definitive efficacy and long-term outcomes require validation in larger Phase III trials.	(92, 93)
MSC-EVs Clinical Trial	COVID-19-induced ARDS (N = 40).	Allogeneic bone marrow MSC-derived extracellular vesicles (EVs); 100 μg/kg, single IV infusion.	**Efficacy:** Improvement in oxygenation; reduced inflammatory markers. **Safety:** No serious adverse events; well-tolerated.	As a “cell-free” alternative, MSC-EVs show promising therapeutic potential and safety, representing a key future translational direction.	(78)
Preclinical/Translational Evidence	*In vitro* & animal models of ALI/ARDS.	MSC-derived extracellular vesicles (MSC-EVs) from various sources.	**Efficacy:** Promising effects on alveolar fluid clearance, barrier repair, immunomodulation, and mitochondrial transfer in models. **Safety (Potential):** EVs are considered to have low immunogenicity and high stability.	Provides a solid mechanistic foundation and rationale for the clinical translation of MSC-EVs.	(25, 26, 71–73)
Combination Therapy Strategies (Emerging)	Preclinical & early clinical studies.	MSCs combined with drugs (e.g., glucocorticoids), gene-edited MSCs, or bioengineered delivery systems.	**Efficacy (Preclinical):** Suggests synergistic enhancement of immunomodulation, cell survival, homing, and tissue repair. **Safety:** Requires further clinical evaluation.	Represents a trend towards personalized and precision therapy, aiming to overcome current limitations through engineering approaches.	(61–70, 72, 81–86)
Challenges & Heterogeneity Statements	Analysis across clinical and preclinical studies.	Highlights variability in cell source, donor, manufacturing, dose, and timing.	**Key Conclusion:** Inconsistent efficacy is linked to patient/disease heterogeneity and lack of standardization.	Future successful translation hinges on establishing standardized cell manufacturing (GMP) and quality control, and conducting biomarker-based precision clinical trials.	(20, 94–98)

Bold values indicate the primary efficacy or safety outcomes reported in each study, including statistically significant findings or key quantitative measures (e.g., mortality, adverse events, or clinical benefit).

The negative outcome of the STAT trial (99) highlights critical biological constraints that may limit MSC efficacy in clinical ARDS. First, the disparity between preclinical success and clinical failure may reflect limitations of animal models, which typically use young, healthy subjects with uniform injuries, failing to recapitulate the complex comorbidities and heterogeneous pathophysiology of human ARDS. Second, the pro-inflammatory milieu in ARDS patients may impair MSC function and survival; high levels of inflammatory cytokines, oxidative stress, and host immune responses can compromise MSCs before they exert therapeutic effects. Third, the optimal therapeutic window may be missed in clinical trials, as MSC-mediated immunomodulation is most effective during the acute phase, whereas patient enrollment often occurs later when structural damage and fibroproliferation predominate. These biological constraints, rather than technical factors alone, likely contribute to the modest and inconsistent clinical benefits observed to date.

### 5.2 Key technical issues in MSCs treatment

Although MSCs have shown great potential in ARDS, their clinical translation remains hampered by several key technical challenges. These challenges span various stages of cell source preparation, delivery, and efficacy evaluation and are key to determining whether MSC therapy can be widely applied from the laboratory to clinical practice.

The foremost challenges currently lie in sourcing, preparing, preserving, and standardizing MSC doses. MSCs can be harvested from diverse tissues, including bone marrow, umbilical cord, adipose tissue, and placenta; however, cells from different origins exhibit marked heterogeneity in proliferative capacity, immunomodulatory function, and differentiation potential, which directly affects the stability and predictability of therapeutic outcomes (94). Even with the same source, individual differences among donors, cell culture conditions, and freezing and thawing processes can alter cell function and phenotype, leading to significant differences between batches (95). This heterogeneity makes it difficult to establish a unified cell quality standard and dose-response relationship. In clinical studies, MSC infusion doses range from millions to billions, and there is a lack of precise dose guidance based on PK/PD, which may be a key reason for inconsistent clinical trial results. Therefore, establishing standardized processes for cell expansion, characterization, and storage that adhere to GMP guidelines is fundamental for ensuring the reproducibility and large-scale application of MSC therapy (96).

The survival rate, targeted migration ability, and long-term safety of cells *in vivo* are core issues that warrant further investigation. Following intravenous infusion, the majority of MSCs become trapped in the pulmonary capillary bed and are rapidly cleared; only a small proportion of cells can effectively home to damaged lung tissue and survive long-term. These low retention and survival rates limit their sustained therapeutic efficacy. Studies have demonstrated that the pulmonary microenvironment in ARDS, characterized by a proinflammatory state, can influence the survival and function of MSCs (97).

The purification and functional validation of EVs are another key factor in the clinical translation of MSC therapy. Many therapeutic effects of MSCs are believed to be mediated through paracrine mechanisms, particularly through the release of EVs (101). MSC-EVs offer advantages like low immunogenicity, high stability, ease of storage, and standardized production, and are considered a promising “cell-free” therapeutic alternative (98). At present, there is a lack of unified, efficient separation and purification techniques, and EVs obtained by different methods exhibit significant differences in purity, yield, and bioactive components. Secondly, it is necessary to strictly define and verify the “identity” and “effectiveness” of MSC-EVs. This includes the proteins it carries and miRNAs. Conduct comprehensive proteomic and transcriptomic analyses of bioactive compounds, such as lipids, and establish causal relationships between these compounds and specific therapeutic functions. Finally, engineering MSC-EVs to enhance their homing ability and therapeutic effect on damaged lung tissue is an important direction for future research. Only by addressing these bottlenecks in production, characterization, and functional validation can MSC-EVs truly become a reliable therapeutic product (102, 103).

### 5.3 Immune rejection and safety issues

The effectiveness of MSC therapy also varies substantially depending on the source, dose, route of administration, and disease state. Meta-analyses of ARDS and COVID-19 have shown beneficial effects of MSCs on survival and clinical outcomes (without increasing adverse events), but the magnitude of benefit has been modest and, in some cases, not statistically significant. Nevertheless, most such trials are small and underpowered, and a lack of standardized manufacturing methods, potency assays for preparation testing, and agreement on optimal dosing schedules all contribute to the inability to construct a confidence interval around a point estimate of efficacy (89, 90) (**Table 1**). Differences in MSC properties, including donor variation and culture conditions, affect cell quality and therapeutic potential (91). However, variability arising from heterogeneity in MSC sources (BM-MSCs, AT-MSCs, and UC/BM-MPPCs), as well as preparation procedures and dosages, complicates safety evaluations across multiple treatment outcomes.

It is necessary to standardize MSC manufacturing for identical efficacy and safety. It is often unclear whether the MSCs tested for immune responses were from the same source and prepared identically. Present clinical studies are conducted with differing sources of MSCs and varying preparation methods, ultimately resulting in differences in cell phenotype and function (**Figure 5**). Preconditioning approaches, including exposure to hypoxia or cytokines, have been investigated to enhance MSC homing, viability, and immunomodulatory activity, thereby potentially increasing therapeutic potential (104). However, such alterations must undergo rigorous safety assessment to eliminate toxicity (**Figure 5**).

Importantly, however, the territory of MSC therapy clinical trials is a moving landscape, and more well-conducted large-scale RCTs are required to confirm safety and efficacy. The majority of current trials are phase I or II trials with few subjects and short follow-up times. For ARDS, meta-analyses indicate that MSCs reduce short-term mortality and are safe, but there is insufficient evidence to demonstrate long-term benefit (92) (**Table 1**). Heterogeneity in MSC sources and production processes has resulted in variable therapeutic outcomes. There is a paucity of clinical evidence from which to draw conclusions, and the need for additional high-quality RCTs is warranted to determine the safety and efficacy. Solving these problems is necessary to translate MSCs therapy into a standard clinical treatment for ARDS and other diseases.

In the preclinical stage, a systematic toxicological and tumorigenic assessment is required. During the clinical research phase, it is imperative to establish a rigorous adverse event monitoring protocol, with a particular focus on infusion-related reactions, infection rates, autoimmune responses, potential tumorigenesis, and the immunogenicity arising from allogeneic transplantation (93). Leveraging modern technologies to monitor the distribution, survival, and functional status of MSCs *in vivo* is of paramount importance. By implementing standardized safety assessment procedures and long-term follow-up systems, we can ensure that MSC therapy not only exerts therapeutic effects but also maximizes patient safety, advancing this cutting-edge therapy towards mature, reliable clinical practice (**Figure 5**).

## 6 Conclusion

The dynamic terrain of MSC therapy for ARDS underscores a complex interplay between immunomodulation and tissue repair in MSCs and suggests that these cells are a promising cell-based therapeutic approach. From an expert perspective, potential well-suited roles for MSCs in the treatment of complex processes in ARDS are considered, given their dual capacity to reduce inflammation and support the beneficial repair of AECs and ECs. This review has shown that the reparative actions of MSCs reside largely in their secretome, which comprises a variety of growth factors and EV-mediated signaling cascades that guide lung tissue repair and restore vascular function.

The interplay between MSCs and ECs, which is responsible for repairing the endothelium and restoring the alveolar-capillary barrier, is a crucial observation from our work. This cell-to-cell cooperation highlights how MSCs not only modify the immune response but can also directly engage in cellular communication, a crucial process for tissue regeneration. The immunomodulatory and reparative roles of MSCs must be balanced to achieve optimal therapeutic effects, as these functions are complementary and contribute to the resolution of lung injury.

Despite promising preclinical findings, significant hurdles hinder the clinical translation of MSC therapy. Key technical challenges include heterogeneity in MSC sources and preparation methods, lack of standardized dosing protocols, and poor *in vivo* survival and homing efficiency (94–97). Long-term safety concerns—such as immunogenicity, tumorigenicity, and aberrant differentiation—also require rigorous evaluation through extended follow-up studies (93). Addressing these biological and technical barriers will be essential for advancing MSC therapy into routine clinical practice.

Furthermore, combining MSC therapy with adjuvant methods, including drug delivery systems, gene-editing approaches, and EV engineering, is a growing trend in ARDS therapy. These combinatorial strategies could increase MSC potency, modulate cell function, and address current deficiencies in cell survival and homing to target organs. Yet this complexity demands extensive mechanistic characterization to identify the specific pathways underlying clinical safety and to ensure consistent applicability.

The discordance between robust preclinical efficacy and modest clinical benefits underscores a critical gap in our understanding of MSC biology within the complex human disease environment. As elaborated in Section 4.1, factors including the limitations of animal models, the hostile inflammatory milieu in patients, and the frequent mismatch between treatment timing and disease stage collectively constrain clinical translation. Addressing these biological barriers—rather than technical factors alone—will be essential for realizing the therapeutic potential of MSCs in ARDS.

MSC repair mechanisms discussed in this article vary in terms of the strength of supporting evidence. Some mechanisms, such as KGF-mediated epithelial repair, Ang-1/Tie2-mediated endothelial stabilization, and mitochondrial transfer-induced anti-apoptosis, have been thoroughly validated across multiple *in vitro* and *in vivo* models of ARDS, forming the core theoretical foundation for MSC therapy. In contrast, other mechanisms, including epigenetic regulation, specific miRNA networks, and lncRNA signaling axes, are primarily derived from studies on other tissues or conducted *in vitro*, and their precise roles and relative contributions within the ARDS lung microenvironment remain to be further elucidated. Clearly distinguishing these different levels of evidence can provide valuable guidance for future research and facilitate the precise refinement of MSC treatment strategies.

Regarding the future of MSC therapy for ARDS, integrating basic biological research with clinical studies can drive further development. Simultaneous exploitation of knowledge across cellular biology, immunology, bioengineering, and clinical sciences will be necessary to elucidate the intricacies of MSC activity and optimize their therapeutic use. By promoting crosstalk between these areas, the field may expedite the optimization of MSC-based regimens that directly impact clinical outcomes.

MSC treatment represents an attractive paradigm shift in how we tackle ARDS, focusing on both immunomodulation and recovery. Although questions remain, recent progress in MSC biology and novel therapeutic advances provide a solid foundation for further investigation and clinical translation. Further research and validation of MSC-based approaches have the potential to revolutionize ARDS therapy, thereby addressing unmet clinical needs and improving survival and quality of life for patients.

## Glossary

ARDS: Acute Respiratory Distress Syndrome
MSCs: Mesenchymal Stem Cells
AECs: Alveolar Epithelial Cells
ECs: Endothelial Cells
VILI: Ventilator-Induced Lung Injury
EVs: Extracellular Vesicles
KGF: Keratinocyte Growth Factor
HGF: Hepatocyte Growth Factor
VEGF: Vascular Endothelial Growth Factor
ENaC: Epithelial Sodium Channel
ALI: Acute Lung Injury
NLRP3: Nucleotide-binding and leucine-rich repeat Family Pyrin Domain Containing Protein 3
GSDME: Gasdermin E
LPS: Lipopolysaccharide
IL-6: Interleukin-6
IL-8: Interleukin-8
ER: Endoplasmic Reticulum
Bip: Binding immunoglobulin Protein
CHOP: C/EBP Homologous Protein
NF-κB: Nuclear Factor Kappa B
FoxM1: Forkhead Box M1
FasL: Fas Ligand
AEC2: type 2 alveolar epithelial
TGF-β: Transforming Growth Factor Beta
Ang-1: Angiopoietin-1
TFAM: Mitochondrial Transcription Factor A
mTORC2: mammalian Target Of Rapamycin Complex 2
PI3K/AKT: Phosphoinositide 3-kinase / Protein Kinase B
S1P: Sphingosine-1-phosphate
MMPs: Matrix Metalloproteinases
IL-10: Interleukin-10
ICAM-1: Intercellular Adhesion Molecule-1
Nrf2: Nuclear Factor Erythroid 2-Related Factor 2
ARE: Antioxidant Response Element
HO-1: Heme Oxygenase-1
SOD: Superoxide Dismutase
ROS: Reactive Oxygen Species
MAPK/ERK: Mitogen-Activated Protein Kinase / Extracellular Signal-Regulated Kinase
EPCs: Endothelial Progenitor Cells
sEVs: small Extracellular Vesicles
HUVECs: Human Umbilical Vein Endothelial Cells
lncRNA: long noncoding RNA
TCA: Tricarboxylic Acid
PDGF: Platelet-Derived Growth Factor
MSC-EVs: Mesenchymal Stem Cell-derived Extracellular Vesicles
CD29: Cluster of Differentiation 29
CD44: Cluster of Differentiation 44
ECM: Extracellular Matrix
IFN-γ: Interferon Gamma
TNF-α: Tumor Necrosis Factor Alpha
PDGF-BB: Platelet-Derived Growth Factor-BB
PDGFRβ: Platelet-Derived Growth Factor Receptor Beta
STAT1: Signal Transducer and Activator of Transcription 1
PGE2: Prostaglandin E2
IDO: Indoleamine 2,3-Dioxygenase
GDF6: growth differentiation factor 6
COVID-19: Coronavirus Disease 2019
BM-MSCs: Bone Marrow Mesenchymal Stem Cells
AT-MSCs: Adipose Tissue Mesenchymal Stem Cells
UC-MSCs: Umbilical Cord Mesenchymal Stem Cells
RCTs: Randomized Controlled Trials
EMT: Epithelial–Mesenchymal Transition
CRISPR/Cas9: Clustered Regularly Interspaced Short Palindromic Repeats / CRISPR-associated protein 9
HLA-G: Human Leukocyte Antigen-G
B2M: Beta-2-Microglobulin
TF/CD142: Tissue Factor / Coagulation Factor III
BMP-9: Bone Morphogenetic Protein 9
CXCR4: C-X-C Chemokine Receptor Type 4.
