# Supplementary material for: Beyond immunomodulation: mechanisms and synergistic strategies of mesenchymal stem cells in promoting alveolar epithelial and endothelial repair in ARDS
Source: Front Immunol. 2026 Jun 12;17:1769714. doi: 10.3389/fimmu.2026.1769714 (PMC13303020; doi:10.3389/fimmu.2026.1769714)
Supplement: Supplementary file 1 [file DataSheet1.docx]

| Abbreviation | Unabridged English name |
| --- | --- |
| ARDS | Acute Respiratory Distress Syndrome |
| MSCs | Mesenchymal Stem Cells |
| AECs | Alveolar Epithelial Cells |
| ECs | Endothelial Cells |
| VILI | Ventilator-Induced Lung Injury |
| EVs | Extracellular Vesicles |
| KGF | Keratinocyte Growth Factor |
| HGF | Hepatocyte Growth Factor |
| VEGF | Vascular Endothelial Growth Factor |
| ENaC | Epithelial Sodium Channel |
| ALI | Acute Lung Injury |
| NLRP3 | Nucleotide-binding and leucine-rich repeat Family Pyrin Domain Containing Protein 3 |
| GSDME | Gasdermin E |
| LPS | Lipopolysaccharide |
| IL-6 | Interleukin-6 |
| IL-8 | Interleukin-8 |
| ER | Endoplasmic Reticulum |
| Bip | Binding immunoglobulin Protein |
| CHOP | C/EBP Homologous Protein |
| NF-κB | Nuclear Factor Kappa B |
| FoxM1 | Forkhead Box M1 |
| FasL | Fas Ligand |
| AEC2 | type 2 alveolar epithelial |
| TGF-β | Transforming Growth Factor Beta |
| Ang-1 | Angiopoietin-1 |
| TFAM | Mitochondrial Transcription Factor A |
| mTORC2 | mammalian Target Of Rapamycin Complex 2 |
| PI3K/AKT | Phosphoinositide 3-kinase / Protein Kinase B |
| S1P | Sphingosine-1-phosphate |
| MMPs | Matrix Metalloproteinases |
| IL-10 | Interleukin-10 |
| ICAM-1 | Intercellular Adhesion Molecule-1 |
| Nrf2 | Nuclear Factor Erythroid 2-Related Factor 2 |
| ARE | Antioxidant Response Element |
| HO-1 | Heme Oxygenase-1 |
| SOD | Superoxide Dismutase |
| ROS | Reactive Oxygen Species |
| MAPK/ERK | Mitogen-Activated Protein Kinase / Extracellular Signal-Regulated Kinase |
| EPCs | Endothelial Progenitor Cells |
| sEVs | small Extracellular Vesicles |
| HUVECs | Human Umbilical Vein Endothelial Cells |
| lncRNA | long noncoding RNA |
| TCA | Tricarboxylic Acid |
| PDGF | Platelet-Derived Growth Factor |
| MSC-EVs | Mesenchymal Stem Cell-derived Extracellular Vesicles |
| CD29 | Cluster of Differentiation 29 |
| CD44 | Cluster of Differentiation 44 |
| ECM | Extracellular Matrix |
| IFN-γ | Interferon Gamma |
| TNF-α | Tumor Necrosis Factor Alpha |
| PDGF-BB | Platelet-Derived Growth Factor-BB |
| PDGFRβ | Platelet-Derived Growth Factor Receptor Beta |
| STAT1 | Signal Transducer and Activator of Transcription 1 |
| PGE2 | Prostaglandin E2 |
| IDO | Indoleamine 2,3-Dioxygenase |
| GDF6 | growth differentiation factor 6 |
| COVID-19 | Coronavirus Disease 2019 |
| BM-MSCs | Bone Marrow Mesenchymal Stem Cells |
| AT-MSCs | Adipose Tissue Mesenchymal Stem Cells |
| UC-MSCs | Umbilical Cord Mesenchymal Stem Cells |
| RCTs | Randomized Controlled Trials |
| EMT | Epithelial–Mesenchymal Transition |
| CRISPR/Cas9 | Clustered Regularly Interspaced Short Palindromic Repeats / CRISPR-associated protein 9 |
| HLA-G | Human Leukocyte Antigen-G |
| B2M | Beta-2-Microglobulin |
| TF/CD142 | Tissue Factor / Coagulation Factor III |
| BMP-9 | Bone Morphogenetic Protein 9 |
| CXCR4 | C-X-C Chemokine Receptor Type 4 |
